# Thyrotropin Stimulates Differentiation Not Proliferation of Normal Human Thyrocytes in Culture

**DOI:** 10.3389/fendo.2016.00168

**Published:** 2016-12-26

**Authors:** Sarah J. Morgan, Susanne Neumann, Bernice Marcus-Samuels, Marvin C. Gershengorn

**Affiliations:** ^1^Laboratory of Endocrinology and Receptor Biology, National Institute of Diabetes and Digestive and Kidney Diseases, National Institutes of Health, Bethesda, MD, USA

**Keywords:** TSH, IGF-1, proliferation, TSHR, TG, cell count, differentiation

## Abstract

Although TSH has been suggested to be a proliferative agent for thyrocytes, the effect of TSH on human thyroid cells remains controversial. In particular, most of the reported studies relied primarily on changes in DNA synthesis but have not included measurement of the number of cells. We argue that only a direct count of cell number, demonstrating classical exponential expansion, serves as a valid measurement of proliferation. Thus, although some data support TSH as a proliferative agent, most do not provide conclusive evidence. To generate conclusive evidence with regard to a proliferative effect of TSH in human thyrocytes, we performed various experiments using primary cultures of human thyrocytes. In contrast to previous reports, TSH [±insulin-like growth factor 1 (IGF-1)] did not induce proliferation of thyrocytes under a variety of different conditions. However, TSH/IGF-1 cotreatment did upregulate thyroid-specific gene expression including *thyroglobulin (TG)* and *TSHR* in a manner consistent with cellular differentiation. Evidence for a proliferative effect of TSH has been used to inform the American Thyroid Association’s guidelines for the management of thyroid cancer patients, which include TSH suppression. While these recommendations are admittedly based on low- to moderate-quality evidence, TSH suppression is still widely used. We present data that question the consensus view that TSH promotes proliferation of human thyrocytes (upon which the American Thyroid Association’s guidelines are based) and suggest that additional studies, including randomized controlled trials, are warranted to address this important clinical question.

The thyrotropin (TSH) receptor (TSHR) plays an important role in thyroid physiology and in the pathophysiology of thyroid diseases. TSHR is a G-protein coupled receptor that serves as the main regulator of development and function of the thyroid. In the adult thyroid gland, TSH, acting through TSHR, stimulates the expression of various thyroid-specific genes that lead to the production and secretion of thyroid hormones.

Herein, we address the question of whether TSH stimulates proliferation of human thyrocytes. Several issues exist with regard to the validity of the current evidence supporting a proliferative effect of TSH in human thyroid cells. Most data concerning the effects of TSH on thyroid cell proliferation have been reported in studies of various thyroid-derived cell lines of rat origin (FRTL-5, WRT, and PC Cl3) as well as primary cultures of dog thyrocytes [reviewed in Ref. (1)]. The consensus view in these models is that TSH increases thyrocyte proliferation and that insulin-like growth factor 1 (IGF-1) or high-dose insulin (activating the IGF-1 receptor) acts synergistically with TSH to promote this effect. The ability to translate findings from these models to human cells and tissues is unclear, and whether TSH stimulates proliferation of human thyroid cells remains controversial (2–4). Westermark et al. (2) studied human thyrocytes in primary monolayer cultures and found that TSH *decreased* [^3^H]thymidine incorporation into DNA and *decreased* cell number. Williams et al. (3) studied human thyroid follicular cells in monolayer and suspension cultures by measuring [^3^H]thymidine incorporation into DNA and by cell cycle progression. In monolayer cultures, they found that TSH had no effect on these parameters, whereas in suspension cultures, TSH, in the presence of growth factors [high doses of insulin or low concentrations of fetal bovine serum (FBS)], increased these parameters. Van Keymeulen et al. (4) reported that TSH and high doses of insulin stimulated DNA synthesis. Overall, reports supporting TSH as a mitogenic agent in human cultures indicate that IGF-1 or insulin is required for this effect.

Importantly, in the majority of these studies, the effects on “proliferation” were demonstrated by measuring incorporation of radiolabeled nucleotides into DNA as a purported measure of DNA synthesis. However, the definition of cell proliferation is “the process that results in an increase of the number of cells… by the balance between cell divisions and cell loss through cell death or differentiation” (5). Therefore, an increase in DNA synthesis is not necessarily an indication of cell proliferation. In our opinion, one must measure an increase in cell number to conclude that cell proliferation has occurred. If no increase in cell number is observed, even if DNA synthesis or the rate of cell cycle progression were increased, cell proliferation has not occurred.

We studied the effects of TSH on proliferation and differentiation of normal human thyrocytes in primary monolayer cultures. This study and experimental protocols were approved by and carried out in accordance with the recommendations of the NIDDK Institutional Review Board. All patients gave written informed consent in accordance with the Declaration of Helsinki. Patient materials were received anonymously with approval of research activity through the Office of Human Subjects Research, National Institutes of Health. Primary cultures of human thyrocytes were established, as described previously (6). Fibroblast contamination of cultures was low and remained as such even following extended propagation in high-serum media. Cells were propagated/maintained in Dulbecco’s modified Eagle’s medium (DMEM), supplemented with 10% FBS, 100 U/mL penicillin, and 10 µg/mL streptomycin (Life Technologies Corp., Carlsbad, CA, USA) at 37°C in a humidified 5% CO_2_ incubator. During continuous propagation in growth medium containing 10% FBS, most human thyrocytes exhibited a doubling time between 72 and 96 h. All cultures, once established, were verified for thyroid-specific gene expression [*thyroglobulin* (*TG*), *thyroperoxidase, sodium-iodide symporter, deiodinase type 2*, and *TSHR*] *via* quantitative RT-PCR. Cells were serum starved using DMEM containing no FBS but 0.1% bovine serum albumin (BSA) for 72 h prior to stimulation. Cultures were stimulated with 10 mIU/mL bovine TSH (bTSH) in combination with 100 ng/mL IGF-1 in DMEM containing 0.1% BSA or 2.5% FBS. After 5 days, cells were harvested, and proliferation was measured by direct cell counting using the Vi-CELL Series Cell Viability Analyzer (Beckman Coulter) and analyzed for levels of *TSHR* and *TG* mRNAs as markers of thyrocyte differentiation by a standard RT-PCR method, as described previously (6).

We initially conducted a series of experiments stimulating thyrocytes with bTSH only in the presence of various levels of FBS. Under no culture conditions did TSH alone elicit an increase in thyrocyte cell number (data not shown). However, IGF-1 is often considered necessary for the mitogenic effects of TSH, particularly in human thyrocytes (1), and we could not exclude the possibility that our negative results were due to this missing permissive factor. Thus, we repeated the original experiments using a combination of TSH and IGF-1 to stimulate proliferation. Figure 1 illustrates the results of three experiments, each utilizing a unique patient donor culture, in which cells were stimulated without or with 10 mIU/mL of bTSH and 100 ng/mL of IGF-1 in medium containing 0.1% BSA or 2.5% FBS; 20% FBS was included to demonstrate the ability of these cultures to proliferate. In these experiments, 2.5% FBS stimulated a 40% increase in cell number over BSA-containing media, whereas 20% FBS stimulated 340% increase in 5 days; this increase, nearly two doublings, is clear evdience of exponential expansion. On the other hand, it is clear that TSH + IGF-1 had no effect on human thyrocyte proliferation in the absence of FBS or in the moderate proliferative environment fostered by the presence of 2.5% FBS. No differences in the proportion of fibroblasts in each culture were observed under any of the studied conditions. Importantly, the unstimulated levels of both *TSHR* and *TG* mRNAs were progressively decreased when the cells were incubated in 2.5% and 20% FBS, a sign of de-differentiation. Moreover, TSH + IGF-1 stimulated increases in *TSHR* and *TG* mRNAs in these cells, a sign of differenitation, and this effect was inhibited by the inclusion of 2.5% FBS. Our previously reported data (6) underline the ability of TSH and IGF-1 to induce differentiation in thyroid cells, and furthermore, demonstrated that cooperativity between TSHR and IGF-1R promotes the upregulation of thyroid-specific functions of human thyrocytes in primary culture. Our findings are consistent with the commonly observed result that differentiation and proliferation are opposing processes—expression of differentiated genes is decreased when cells are proliferating, and upregulation of cell-specific, differentiated gene expression is not accompanied by stimulation of cell proliferation.

**Figure 1:**
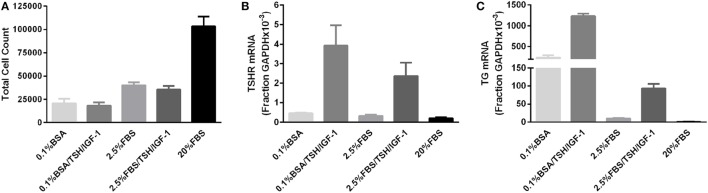
**TSH in combination with insulin-like growth factor 1 (IGF-1) does not promote proliferation of human thyrocytes in primary culture but induces differentiated gene expression**. **(A)** Human thyrocytes were seeded, allowed to attach overnight, then arrested for 3 days in 0.1% bovine serum albumin-containing Dulbecco’s modified Eagle’s medium. Cells were then stimulated with the indicated doses of TSH alone or in combination with 100 ng/mL IGF-1. The cell count at the time of stimulation was 25,500 ± 6,245 cells. Cells were harvested and counted after 5 days of treatment. Under no culture conditions did TSH alone elicit an increase in thyrocyte cell number (data not shown). A portion of the sample was also analyzed for **(B)** *TSHR* and **(C)** *thyroglobulin* mRNA expression using quantitative PCR. Each experiment contained biological duplicates. Data are expressed as mean ± SEM, *n* = 3 patient donors.

In addition to the data presented here, we have performed similar experiments in cultures derived from a total of 10 patient donors utilizing a variety of conditions, including but not limited to various additional concentrations of FBS, TSH doses ranging from 0.01 to 100 mIU/mL, TSH or IGF-1 in the absence of the other ligand, and time points ranging from 3 to 14 days of stimulation (data not shown). No condition demonstrated a proliferative effect of TSH on human thyrocytes in culture. In fact, following 14 days of stimulation, virtually all cultures in 20% FBS were completely confluent (having undergone 4–5 doublings) and contact inhibited, while cultures in BSA or low serum (with or without additional factors including TSH) had not undergone even a single doubling. Also not shown here, we have not found any proliferative effect of TSH (±IGF-1) on three other human thyroid culture models: the immortalized normal human thyrocyte line Nthy-ori 3.1 (7) and two patient-derived follicular thyroid cancer lines, FTC-133 (8) and ML-1 (9). This held true even when the TSH receptor was overexpressed *via* adenoviral infection as per Ref. (10), although infection did significantly increase TSH-mediated cAMP generation in these cells. Thus, TSH promotes differentiation but does not stimulate proliferation of human thyrocytes in culture.

Evidence supporting a proliferative effect of TSH has been used to inform the American Thyroid Association’s guidelines for the management of thyroid cancer patients, which include the suppression of TSH to prevent disease recurrence and/or progression (11). The standard of care for differentiated thyroid cancer consists of total thyroidectomy, followed by radioiodine therapy (RAI) in the subset of patients with intermediate and high-risk disease. Long-term management includes levothyroxine treatment with the dose adjusted to achieve specific TSH values based on risk level. Currently, TSH suppression below 0.1 mIU/L is recommended for high-risk patients (Strong recommendation, *Moderate-quality evidence*), 0.1–0.5 mIU/L for intermediate-risk patients (Weak recommendation, *Low-quality evidence*), and 0.5–2 mIU/L for low-risk patients (Weak recommendation, *Low-quality evidence*). As indicated in the guidelines, these recommendations are based on low- or moderate-quality evidence–predominantly retrospective studies with limited numbers of patients. Regardless, TSH reduction to these levels is a recommended component of thyroid cancer treatment, although full TSH suppression is now recommended only for high-risk patients. TSH suppression may result in undesirable side effects and lower quality of life for the patient while providing a questionable clinical benefit (12).

Although we would not conclude that the results presented herein have direct relevance to the effects of TSH in humans, we suggest that the general view that TSH is a human thyrocyte proliferating factor be reconsidered. Further clarification, including double-blind clinical trials leading to high-quality evidence, is necessary to definitively address these concerns.

## Ethics Statement

This study was carried out in accordance with the recommendations of the NIDDK Institutional Review Board with written informed consent from all subjects. All subjects gave written informed consent in accordance with the Declaration of Helsinki. Patient materials were received anonymously with approval of research activity through the Office of Human Subjects Research, National Institutes of Health.

## Author Contributions

All authors made significant contributions to the experiments and manuscript.

## Conflict of Interest Statement

The authors have no conflicts to report. This research was conducted in the absence of any commercial or financial relationships that could be construed as a potential conflict of interest.
